# Diagnostic values of BALF metagenomic next-generation sequencing, BALF real-time PCR and serum BDG for *Pneumocystis jirovecii* pneumonia in HIV-infected patients

**DOI:** 10.3389/fmicb.2024.1421660

**Published:** 2024-09-20

**Authors:** Qianhui Chen, Xiaoping Chen, Pingzheng Mo, Liangjun Chen, Qian Du, Wenjia Hu, Qunqun Jiang, Zhongwei Zhang, Yongxi Zhang, Qinglian Guo, Yong Xiong, Liping Deng

**Affiliations:** ^1^Department of Infectious Diseases, Zhongnan Hospital of Wuhan University, Wuhan, China; ^2^AIDS Research Center, Wuhan University, Wuhan, China; ^3^Department of Clinical Laboratory, Zhongnan Hospital of Wuhan University, Wuhan, China

**Keywords:** metagenomics next-generation sequencing, Real-Time PCR, *Pneumocystis jirovecii* pneumonia, HIV/AIDS, diagnosis

## Abstract

**Introduction:**

This study aimed to assess the diagnostic values of bronchoalveolar lavage fluid (BALF) real-time polymerase chain reaction (PCR) and BALF metagenomic next-generation sequencing (mNGS) for Pneumocystis jirovecii pneumonia (PJP) in patients infected with human immunodeficiency virus (HIV).

**Methods:**

A total of 99 HIV-infected PJP patients and 61 HIV-infected patients diagnosed with non-PJP pneumonia between March 2019 and December 2022 were enrolled. *P. jirovecii* and multiple other co-pathogens detected in BALF by mNGS were analyzed. The clinical final diagnosis was employed as a benchmark. We compared the diagnostic performance of mNGS in PJP with serum BDG and BALF real-time PCR. The mixed infections detected by mNGS and modifications of antimicrobial treatment were also analyzed.

**Results:**

The sensitivity of mNGS test of BALF samples reached 85.86%, which was significantly higher than serum BDG (39.39%, *P* < 0.001). The sensitivity of BALF *P. jirovecii* PCR (84.85%) was similar with mNGS (*P* > 0.05). The specificity of mNGS (100%) was also same as PCR (100.0%), and superior to serum BDG (88.52%, *P* < 0.001). Besides, mNGS performs remarkably well in identifying co-pathogens of PJP patients infected with HIV. In addition to *P. jirovecii*, 82 cases (82.83%) of other co-pathogens were identified based on mNGS. Moreover, thirty-four patients (34.34%) increased therapeutic dose of trimethoprim-sulfamethoxazole (TMP-SMZ) based on BALF *P. jirovecii* PCR. Based on the mNGS results, initial antimicrobial treatment was modified in 86.87% (86/99) of PJP patients.

**Conclusion:**

BALF mNGS and real-time PCR are two powerful techniques for rapid diagnosis of PJP with high specificity and sensitivity. Moreover, the benefit of mNGS is that it may identify other organisms besides PJP and it may benefit proper and prompt treatment.

## 1 Introduction

For *Pneumocystis jirovecii* pneumonia (PJP), caused by *Pneumocystis jirovecii* (*P. jirovecii*), is one of the most common opportunistic infections worldwide in the immunocompromised population (Nasr et al., 2022). In human immunodeficiency virus (HIV) infected individuals, PJP mainly occurs in people with peripheral blood CD4^+^ lymphocytes (CD4^+^ T count) of less than 200 cells/μL or 14% of all lymphocytes (Atkinson et al., 2021). The common signs and symptoms of PJP in acquired immunodeficiency syndrome (AIDS) are the subacute onset of exertional dyspnea, dry cough and fever, and the subacute course over numerous days or even weeks (Nasr et al., 2022). However, the deterioration can also additionally arise rapidly, with respiratory failure being the most frequent (Nasr et al., 2022). The incidence of PJP in HIV-infected patients has decreased dramatically following the introduction of antiretroviral therapy (ART) in resource-rich settings. However, as a major health problem for AIDS patients, PJP is still a significant cause of mortality and morbidity in recent decades (Miller et al., 2013) in areas with limited resources. Hence, early and rapid diagnosis plays an important role in antifungal therapy, improving prognosis, and decreasing mortality.

Delayed or missing diagnosis of PJP may compromise the early initiation of appropriate treatment, thereby fueling the risk of poor outcomes (Cillóniz et al., 2019). However, it is difficult to confirm the diagnosis of PJP. One point is the un-specific respiratory symptom. The clinical manifestations of PJP vary greatly among individuals, and may even be insidious, making the clinical diagnosis difficult, especially for AIDS patients during the period of COVID-19 pandemic (Nasr et al., 2022; Sokulska et al., 2015). Lack of understanding of the disease is prone to missed diagnosis and misdiagnosis. Another point is the lack of an appropriate culture system in vitro for *P. jirovecii* (Nasr et al., 2022). Besides, the final diagnosis of PJP previously mainly depends on the etiological evidence of cysts or trophozoites of the *P. jirovecii*. However, microscopic detection depends primarily on the observer’s experience and is time-consuming (Nasr et al., 2022). Currently, (1,3)-β-D-glucan (BDG) test was rapid and has relatively high negative predictive value, but it was unspecific (Salzer et al., 2018) and has a wide range of false positive sources. Serum lactate dehydrogenase (LDH) is also one of laboratory features associated with PJP. However, An elevated LDH level may be detected as a result of other lung diseases and/or various extrapulmonary disorders (Esteves et al., 2015). In addition, quantitative real-time polymerase chain reaction (PCR) is an important additional diagnostic method due to its high sensitivity and specificity (Salzer et al., 2018). But it still does not widely replace previous methods in areas with limited resources now. In addition, mixed infections are common in PJP patients with HIV infection, however, the diagnostic value of *P. jirovecii* PCR is limited in identifying mixed infections. Therefore, it is urgent to explore more accurate, efficient and comprehensive microbiological diagnostic tools for PJP in HIV-infected patients.

Metagenomic Next-Generation Sequencing (mNGS) has been recognized as a promising technique for clinical microbiological identification, which has the advantages of unbiased, fast and high sensitivity (Zhang et al., 2019; Chen et al., 2023; Goldberg et al., 2015). It can simultaneously detect a variety of infectious agents, including bacteria, viruses, parasites and fungi (Chen et al., 2023; Goldberg et al., 2015). It is worth noting that the diagnostic value of mNGS for PJP in HIV-infected patients still calls for further exploration.

Thus, this study aimed to compare the diagnostic values of bronchoalveolar lavage fluid (BALF) mNGS, real time PCR and serum BDG for PJP and evaluate the diagnostic values of BALF mNGS for co-pathogen infections of PJP in HIV/AIDS individuals.

## 2 Materials and methods

### 2.1 Study design and subjects

We consecutively enrolled 160 HIV-infected patients who were hospitalized to the Infection Department of Zhongnan Hospital of Wuhan University (Wuhan, China), from between March 2019 and December 2022 in this case-control study. Inclusive criteria: (1) HIV infection; (2) concomitant with fever or dry cough, dyspnea, chest tightness; (3) The serum BDG and lactate dehydrogenase (LDH) results were obtained within 7 days of admission; (4) BALF samples were collected for mNGS and *P. jirovecii* real-time PCR; (5) other common laboratory features such as BALF culture and GM test were performed for pulmonary infection caused by pathogens other than *P. jirovecii*. The non-PJP patients who were admitted to our department withing the same time duration were consecutively enrolled as the control group. The clinical comprehensive diagnosis of PJP or non-PJP was made follow the revised EORTC/MSGERC 2020 criteria of invasive fungal infection (Donnelly et al., 2020), based on clinical symptoms, laboratory findings, microbiologic examination, chest radiology and treatment response. To reduce the biases, they were made by two senior expert pulmonologists after discussion with the medical team. The details were as follows: (1) accompanied by fever or dry cough, shortness of breath; (2) multiple ground-glass interstitial exudation, reticulate or consolidated shadows in both lungs were found by Chest computed tomography (CT); (3) positive serum BDG results for twice; (4) *P. jirovecii* cysts (and/or trophozoites) were microscopically identified following Gomori methenamine silver staining. The clinical diagnosis was made if the previously indicated items (1–3) were met, and confirmed diagnosis was made if items (1–4) were met.

**FIGURE 1:**
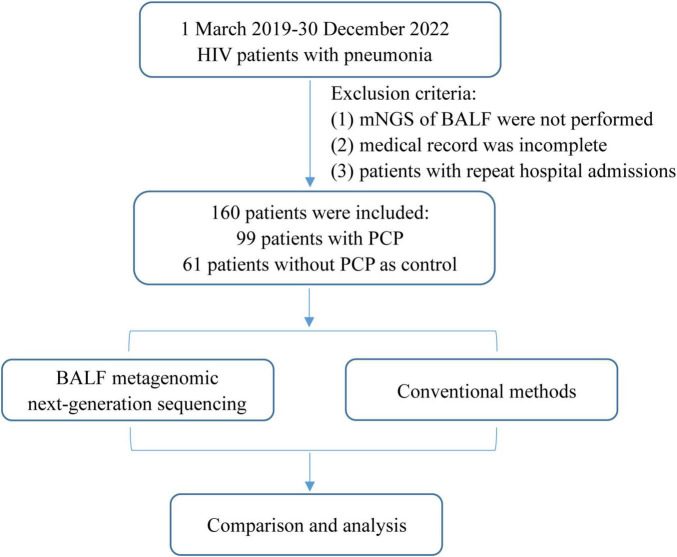
Flowchart of case selection. A total of 160 HIV-infected cases in the Infection Department were selected for further analysis. mNGS, metagenomic next-generation sequencing; BALF, bronchoalveolar lavage fluid; PJP, *Pneumocystis jirovecii* pneumonia.

Patients who meet the subsequent criteria were excluded: (1) The mNGS of BALF were not performed; (2) medical record was incomplete; (3) patients with repeat hospital admissions (Figure 1).

### 2.2 BALF collection and etiological diagnosis

After local anesthesia with lidocaine, BALF was performed by experienced bronchoscopists following standard procedures in Zhongnan Hospital of Wuhan University. The sampling locations were selected based on the chest CT images. After multiple injections of the same amount of normal saline into the affected bronchial segment, BALF was aspirated under negative pressure. BALF was collected for detection according to the guidelines (Meyer et al., 2012).

Meanwhile, the BALF and peripheral blood samples were simultaneously submitted for etiological examination. In this study, microbiologic tests for *P. jirovecii* included serum BDG, LDH, PCR and mNGS. In addition, other common laboratory tests, such as BALF culture, GM test, Gram staining, CMV-DNA and DNA of mycobacterium tuberculosis (TB), were performed to find out whether *P. jirovecii* was co-infection with other pathogens in lung.

### 2.3 DNA Extraction, DNA library construction

Briefly, BALF (600 μL per sample) was taken and mixed with lysozyme and glass beads, vortex for 30 min (2800–3200 rpm). Then, supernatant was obtained (300 μL per sample). DNA was extracted from 300 μl of stored plasma using the TIANamp Micro DNA kit (Tiangen Biotech) following the manufacturer’s operational manual. The extracted DNA were used for the construction of DNA libraries. The total DNA was fragmented by Bioruptor^®^ Pico (Diagenode, Belgium) to 150 bp. Ploy A tail was added to the fragmented DNA for end-repair purposes. Samples-specific adapters were ligated overnight according to the instruction of manufacture (RM 0438, BGI Biotechnology (Wuhan) Co., Ltd) and polymerase chain reaction (PCR) amplification was conducted. DNA libraries were purified with magnetic beads purification strategy using MGIEasy DNA purification kit (MGI, China). Agilent 2100 Bioanalyzer (Agilent Technologies, Carlsbad, California) and Qubit 2.0 (Invitrogen, USA) were used for quality control of the DNA libraries. Qualified libraries (concentration > 2 ng/μL, medium sequence size range 200 to 300 bp) with more than 20 million reads were generated from each sample.

### 2.4 Sequencing

The generated double-stranded DNA library was converted into single-stranded circular DNA through DNA degradation and cyclization. DNA nanospheres (DNBs) were then generated by rolling circle amplification (RCA) techniques. Qualified DNBs were loaded onto a chip and then subjected to 20M 50 bp single-end sequencing on the BGISEQ-2000 platform (BGI-Shenzhen, Shenzhen, China). Microbial-free Hela cells were used as the negative control. The positive control was made of known microbial population. Both controls went through DNA extraction, library construction and sequencing process along with the samples to be tested.

### 2.5 Bioinformatic analysis

Fastq files were obtained, and the quality check of Fastq data was performed by an in-house software with three quality controlling steps: (1) removal of reads less than 35bp; (2) removal of reads which had more than 30% bases of phred 33 scores less than 5; (3) removal of reads containing more than 10 unspecified bases. After that, Burrows-Wheeler Aligner software (v0.7.10-r789) was used for computational subtraction of human host sequences mapped to the human reference genome (hg19) (parameter: bwa mem -k 19 -t 8 -Y -h 10000) (Li and Durbin, 2010). The remaining data by removal of low-complexity reads were classified by simultaneously aligning to four Microbial Genome Databases (PM-seq^®^ commercial database) consisting of bacteria, fungi, viruses, and parasites. The classification reference databases were downloaded from NCBI.^1^ RefSeq contains 4,945 whole genome sequence of viral taxa, 6,350 bacterial genomes or scaffolds, 1064 fungi related to human infection, and 234 parasites associated with human diseases. The mismatch rate was defined as the number of aligned reads with a specific mismatch pattern over the total number of aligned reads. During the alignment procedure, up to 1 mismatch was allowed. Data analytics algorithms were used to exclude the microorganisms that were not significantly related to clinical infection. The uniquely mapped reads that were described as stringently mapped reads number (SMRN) were calculated. The coverage ratio and depth of each microorganism were calculated using BEDTools (v2.29.2).

The existence of pathogens were determined according to the following rules (Miao et al., 2018):

(1) Viruses, bacteria and parasites: mNGS identified microbes (species level) as confirmed pathogens if literature has reported the coverage rate or the pathogenicity was at least 10-fold greater than that of any other microbes that were identified in clinical samples (Langelier et al., 2018).

(2) Fungi: mNGS identified a microbe (species level) as pathogens when the coverage rate scored 5-fold higher than that of any other fungus because of its low biomass in DNA extraction (Bittinger et al., 2014; Schlaberg et al., 2017).

### 2.6 Process of pneumocystis real-time PCR

The sequence of the primer pairs used was described in one previous study (Lu et al., 2022). The conditions used were: 40 cycles of denaturation for 5 min at 94 C; annealing for 15 s at 94 C; and extension for 30 sec at 60 C. PCR was performed using ABI 7500 (Applied Biosystems). Each experimental run incorporated positive, negative, and extraction controls. The cycle threshold (CT) value was documented for positive samples in each run. The significantly increased fluorescence signal, the S-shaped amplification curve, with a CT value ≤ 40 were determined as positive.

### 2.7 Statistical analysis

The statistical analysis was conducted using SPSS 25.0 software (IBM Corp., Armonk, NY, USA). Median and interquartile ranges were used to present numerical variables, while counts and percentages were used for nominal variables. Fisher’s exact test was employed to compare count data across groups in our study. The Mann-Whitney U test was employed to compare the differences of numerical variables between PJP and non-PJP groups, and the chi-square test was utilized for nominal variables. Sensitivity, specificity, positive predict value (PPV) and negative predict value (NPV) along with 95% confidence intervals (CI) were calculated utilizing the clinical composite diagnosis as the reference standard. Statistical significance was determined at *P* < 0.05. The kappa statistic was used to measure the level of agreement between PCR and mNGS for diagnosing *P. jirovecii* infection.

## 3 Results

### 3.1 Clinical characteristics and laboratory results

In our research, we included 160 individuals diagnosed with HIV, comprising 99 individuals in the cases group (PJP group) and 61 individuals in the control group (non-PJP group). The clinical data and results were summarized in Table 1. The median ages (49 vs. 48 years old, *P* = 0.786) of these two cohorts were similar. Most participants were male, and no difference was found in the gender composition ratio between these two groups (84.8% VS 91.8%, *P* = 0.196). The common symptoms observed in PJP patients including fever (71.7%), cough (69.7%), dyspnea (38.4%) and chest tightness (40.4%), but the composition ratios were similar to that of control group.

**TABLE 1:** Clinical characteristics, laboratory findings and radiologic features of PJP and non-PJP patients on admission.

Characteristics (median [IQR] or n [%])	PJP patients (*n* = 99)	Non-PJP patients (*n* = 61)	*p*-value
Age (years)	49 (36–57)	48 (39–53.5)	0.786
Male	84 (84.8%)	56 (91.8%)	0.196
**Clinical symptoms**			
Fever	71 (71.7%)	38 (62.3%)	0.214
Cough	69 (69.7%)	40 (65.6%)	0.587
Dyspnea	38 (38.4%)	19 (31.1%)	0.353
Chest tightness	40 (40.4%)	20 (32.8%)	0.334
**Laboratory test**			
White blood cell count, × 10^9^/L	3.9 (3–6.1)	4.5 (3.145–5.755)	0.508
Neutrophil count, × 10^9^/L	2.55 (1.8–4.67)	3.02 (1.78–4.235)	0.598
Lymphocyte count, × 10^9^/L	0.63 (0.42–1.00)	0.8 (0.38–1.12)	0.463
Hg, g/L	106 (90–118)	103 (83–120.35)	0.210
Platelet count, × 10^9^/L	174 (119–243)	210 (138–279.5)	0.115
Alanine aminotransferase, U/L	22 (13–44)	17 (13–31)	0.175
Aspartate aminotransferase, U/L	29 (21–53)	27 (18.5–42.5)	0.127
Albumin, g/L	30.3 (27–34.9)	29.9 (26.35–35.45)	0.617
Globulin, g/L	36.7 (32.5–44.7)	34.1 (29.85–44.55)	0.173
Creatinine, μmol/L	64.8 (59.5–74.5)	66.2 (56–77.85)	0.884
CD4^+^ T cell (cells/μl)	29 (11–77)	86 (29.5–257.25)	0.001*
Lactate dehydrogenase, U/L	300 (229.5–394.5)	205.5 (159.75–316)	0.001*
ESR, mm/h	65.5 (34.25–91.75)	51.5 (25–93)	0.611
C-reactive protein, mg/L	33.19 (11.55–78.65)	32.8 (16.6–86.1)	0.528
Interleukin-6, pg/mL	19.7 (9.525–39.36)	32.2 (10.39–80.375)	0.156
Procalcitonin, ng/mL	0.06 (0.05–0.26)	0.07 (0.05–0.5)	0.467
Chest CT abnormalities	98 (99.0%)	60 (98.4%)	1
Ground-glass opacity	45 (45.9%)	5 (8.3%)	< 0.001**
Patchy shadowing	59 (60.2%)	36 (52.2%)	0.302
Consolidation	9 (9.2%)	12 (20%)	0.052
Pleural effusion	15 (15.3%)	17 (28.3%)	0.048*
Cavitation	6 (6.1%)	3 (5.0%)	0.768
Nodule	17 (17.3%)	14 (23.3%)	0.358

*P < 0.05 and **P < 0.001.

In HIV-infected patients, no significant statistical difference was observed between PJP group and non-PJP group in the following laboratory test results, including white blood cell count, lymphocyte count, neutrophil count, platelet count, hemoglobin, albumin, globulin, creatinine, alanine aminotransferase, aspartate aminotransferase, C-reactive protein, erythrocyte sedimentation rate (ESR), procalcitonin and interleukin-6 (all *P* > 0.05). However, the median serum LDH in PJP patients was 300 U/L, significantly higher than the non-PJP patients (205.5 U/L, *P* = 0.001). In the PJP group, the median count of CD4^+^ T cell was 29 cells/μl, which was notably lower compared to the non-PJP group (86 cells/μl, *P* = 0.001).

As for chest CT, the most common radiological manifestation in PJP patients were ground-glass opacity (45.9%) and patchy shadowing (60.2%). And the ratio of ground-glass opacity in PJP patients was significantly higher than that in the non-PJP individuals (*P* < 0.001). The ratio of pleural effusion in PJP patients was significantly lower than that in the non-PJP individuals (*P* = 0.048). No difference was found in the patchy shadowing (*P* = 0.302), consolidation (*P* = 0.052), cavitation (*P* = 0.768) and nodule (*P* = 0.358) composition ratio between these two groups respectively.

### 3.2 Comparison of diagnostic performance among mNGS, PCR in BALF, and serum BDG test in PJP of HIV-infected patients

As shown in Table 2, mNGS of BALF and other diagnostic methods, including PCR and serum BDG, were performed in all patients. Our findings illustrated that the CT value of all cases with PJP calculated by mNGS was less than 40, indicating that the mNGS method for detecting PJP was reliable. Herein, we evaluated the diagnostic sensitivity and specificity by utilizing the clinical composite diagnosis as the reference standard. The sensitivity of mNGS was 85.86% (95% CI, 77.07–91.78), which was significantly higher than that of serum BDG [39.39% (95% CI, 29.87–49.75), *P* < 0.001]. The sensitivity of BALF PJP PCR [84.85% (95% CI, 75.92–90.99)] was similar to mNGS, and no significant difference was found between these two groups (*P* > 0.05). In addition, The Kappa value (0.636) showed a relatively high consistency between PCR and mNGS for diagnosing P. jirovecii infection (*P* < 0.001) (Table 3).

**TABLE 2:** Diagnostic performance of mNGS, PCR in BALF, and serum BDG test for HIV-infected PJP and Non-PJP patients.

Methods	PJP cohort	Non-PJP cohort	Sensitivity (95% CI)	Specificity (95% CI)	PPV (95% CI)	NPV (95% CI)
**mNGS**						
+	85	0	85.86%	100%	100%	81.33%
−	14	61	(77.07–91.78)	(92.62–100)	(94.61–100)	(70.33–89.06)
**PJP PCR**						
+	84	0	84.85%	100%	100%	80.26%
−	15	61	(75.92–90.99)	(92.62–100)	(94.55–100)	(69.23–88.18)
**BDG**						
+	39	7	39.39%**	88.52%**	84.78%**	47.37%**
−	60	54	(29.87–49.75)	(77.17–94.88)	(70.52–93.16)	(38.02–56.90)

mNGS, metagenomic next-generation sequencing; PCR, polymerase chain reaction; BDG, serum (1,3)-β-D-glucan, GM, Galactomannan; CI, confidence intervals; PPV, positive predictive value; NPV, negative predictive value.

*P < 0.05 and **P < 0.001 when comparing mNGS total with serum BDG.

**TABLE 3:** Evaluation of the consistency of PCR and mNGS for detecting *P. jirovecii*.

		mNGS		Total	Kappa value	*P*-value
		+	−			
PCR	+	70	14	84	0.636	< 0.001
	−	15	61	76		
	Total	85	75	160		

mNGS, metagenomic next-generation sequencing; PCR, polymerase chain reaction.

In addition, mNGS showed the good specificity of 100% (95% CI, 92.62–100), which was comparable to PCR [100% (95% CI, 92.62–100] and significantly higher to serum BDG [88.52% (95% CI, 77.17–94.88), *P* < 0.001] (Table 2). Furthermore, mNGS also exhibited an excellent positive predict value (PPV) of 100% (95% CI, 94.61–100) and negative predict value (NPV) of 81.33% (95% CI, 70.33–89.06), both of which were significantly higher to those of serum BDG (*P* < 0.001) (Table 2).

### 3.3 Mixed infections and co-pathogens identified by mNGS

**FIGURE 2:**
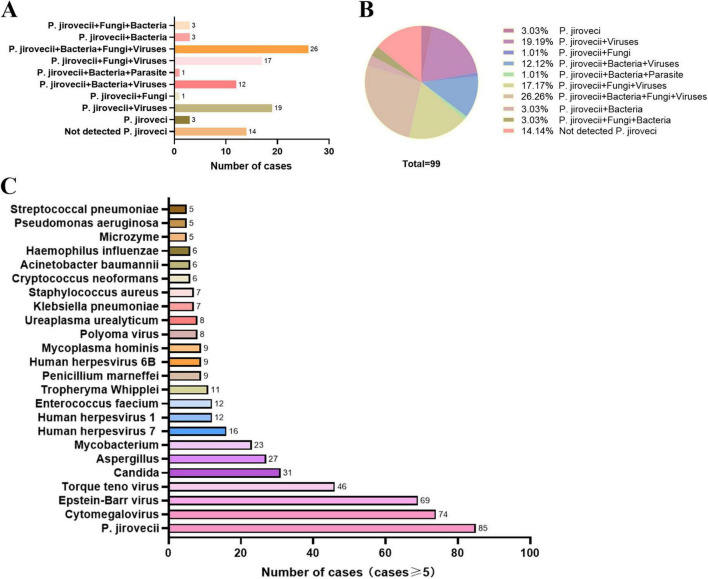
Mixed infections and co-pathogens identified by mNGS in 99 PJP patients with HIV infection. **(A)** number of PJP patients with mixed infections; **(B)** proportion of different types of mixed infection among PJP patients; **(C)** number of PJP patients infected with various co-pathogens. mNGS, metagenomic next-generation sequencing; PJP, *Pneumocystis jirovecii* pneumonia; P. *jirovecii*, *Pneumocystis jirovecii*.

PJP patients infected with HIV often suffer mixed infection due to their immune insufficiency. Numbers of putative mixed infection cases in PJP patients detected by mNGS are showed in Figure 2. Due to the constraints inherent of the DNA detection procedure, only DNA viruses were identified in this study. In addition to *P. jirovecii*, 82 cases (82.83%) of other co-pathogens were identified based on mNGS. *P. jirovecii*-virus-fungi-bacteria co-infection and *P. jirovecii*-virus co-infection were the most common co-pathogens observed in PJP group, which were identified by mNGS in 26 (26.26%) and 19 (19.19%) of 99 patients respectively (Figures 2A, B). Besides, 17 cases (17.17%) of *P. jirovecii*-virus-fungi co-infection were identified by mNGS (Figures 2A, B). In addition, as shown in Figure 2C, *Cytomegalovirus* (74 cases) and *Epstein-Barr virus* (69 cases) were the top two common concurrent pathogens. Moreover, *Torque teno virus* (46 cases), *Candida* (31 cases) and *Aspergillus* (27 cases) were also the common concurrent pathogens. Of note, *Mycobacterium* (23 cases), *Penicillium marneffei* (9 cases) and *Cryptococcus neoformans* (6 cases) were detected in PJP patients. Interestingly, 12 cases were found with a positive GM test of BALF in the 99 PJP patients, including *Penicillium marniffei* combined with *Aspergillus* in four patients, *Penicillium marniffei* in one patient and aspergillus in two patients detected by BALF mNGS (Table 4), which showed that the sensitivity of BALF mNGS was markedly higher than that of GM test for *Aspergillus* and *Penicillium marniffei*. In addition, these results indicated that more attention should be paid to concurrent infections of PJP with tuberculosis, aspergillus, *Penicillium marneffei* and/or *Cryptococcus*.

**TABLE 4:** Detection of *Aspergillus* and *Penicillium marneffei* in patients with positive GM test by BALF mNGS in HIV-infected PJP patients.

	mNGS (31/99)	Positive GM test (12/99)
*Penicillium marneffei*	4	1
*Aspergillus*	22	2
*Penicillium marneffei* and *Aspergillus*	5	4
Total	31	7

mNGS, metagenomic next-generation sequencing; BALF, bronchoalveolar lavage fluid.

### 3.4 Influence of mNGS on antimicrobial treatment for PJP in individuals with HIV infection

Life Records of antimicrobial therapy against *P. jirovecii* and other pathogens duration of hospital stay were obtained from 99 HIV-infected PJP patients. Thirty-four patients (34.34%) increased therapeutic dose of trimethoprim-sulfamethoxazole (TMP-SMZ) based on BALF *P. jirovecii* PCR. According to the results of mNGS, initial antimicrobial treatment was altered in 86.87% of patients. And they were discharged from hospital with improvement finally. 37.37% of patients did not receive a therapeutic dose of trimethoprim-sulfamethoxazole (TMP-SMZ) until the mNGS results were reported. Fifty cases (50.51%) were added with other antifungal drugs, including amphotericin B (AMB) (8.08%), voriconazole (19.19%), fluconazole (14.14%), caspofungin (6.06%) and itraconazole (3.03%). 24 (24.24%) cases were added with antibacterial drugs, and 24 (24.24%) cases were added with antiviral drugs. In addition, 22 (22.22%) cases were added with anti-mycobacterial drugs according to mNGS results. All the adjustments were shown in Table 5.

**TABLE 5:** Impact of mNGS on antimicrobial treatment on PJP patients with HIV infection.

Adjustments	Cases (n [%])
Total adjustments	86 (86.87%)
Add itraconazole	3 (3.03%)
Add TMP-SMZ	37 (37.37%)
Add amphotericin B	8 (8.08%)
Add caspofungin	6 (6.06%)
Add fluconazole	14 (14.14%)
Add voriconazole	19 (19.19%)
Add anti-mycobacterial drugs	22 (22.22%)
Add antiviral drugs	24 (24.24%)
Add antibacterial drugs	24 (24.24%)

TMP-SMZ, trimethoprim-sulfamethoxazole.

## 4 Discussion

Herein, the diagnostic values of BALF mNGS, PCR and BDG for PJP in HIV-infected patients were evaluated in our study. And our findings revealed that mNGS exhibited an excellent sensitivity and specificity in the diagnosis of PJP. Moreover, mNGS demonstrated apparent advantages in detecting co-pathogens in mixed pulmonary infections.

PJP was initially identified in children with compromised immune system during World War II and became widely recognized among adults with HIV infection (Bateman et al., 2020). Currently, PJP is still one of the major AIDS-related opportunistic infection in areas with limited resources (Nasr et al., 2022). LDH is a ubiquitous intracellular enzyme expressed in nearly all types of tissues (Tasaka et al., 2007). In HIV-infected patients, we found that the level of LDH in PJP patients was remarkably higher than the non-PJP patients. Previous study had also shown that LDH is elevated in > 90% of HIV-infected PJP patients. Vogel et al. found that 100% of the HIV-positive patients exhibited elevated levels of LDH, whereas only 63% in HIV-negative patients, suggesting that this marker may be more effective in identifying PJP in individuals with HIV infection (Vogel et al., 2011). However, there have been reports indicating a correlation between LDH levels and oxygenation as well as neutrophil levels (Tasaka et al., 2007; Nakamura et al., 2009). Thus, LDH levels could be indicative of the underlying lung inflammation and injury, rather than being specific to PJP diagnosis.

Patients with immune dysfunction are prone to *P. jirovecii* infection. Currently, in cases of HIV infection, it is recommended that all individuals receive prophylactic therapy when the CD4+ T cell count falls below 200 cells per millimeter (Cillóniz et al., 2019; Kaplan et al., 2002; Limper et al., 2011). The incidence of PJP decreased significantly among individuals with HIV after ART and prophylactic therapy of PJP in resource-rich settings. In our study, the PJP group showed the lower CD4^+^ T cell count compared with non-PJP group, which suggested that HIV positive patients had severe immune deficiency when PJP was confirmed. Significantly, patients may develop PJP despite being on prophylactic therapy (Bateman et al., 2020; Kofteridis et al., 2014), so early screening of HIV-infected patients should still be strengthened after extensive ART treatment, especially with high clinical suspicion.

PJP remains a high mortality when it does occur. In HIV-positive patients, the hospital survival for PJP ranges from 7 to 20% (Bienvenu et al., 2016; Roux et al., 2014; Su et al., 2008). Thus, it is crucial for diagnosis and treatment of PJP in its early stages. However, *P. jirovecii* is extremely difficult to culture. Currently, PCR and serum BDG are employed in the microbiologic identification of PJP (Bateman et al., 2020). mNGS is a promising molecular biology technique for pathogen identification, offering notable benefits in terms of speed, sensitivity, and accuracy (Wu et al., 2020). The hospital in our study treats HIV patients in Wuhan, as well as patients transferred from the surrounding area. And all the test items in this study could be routinely carried out in the hospital. In our experiment, comparing these detection methods, it was observed that the sensitivity of mNGS was similar to PCR, and significantly higher than serum BDG. The Kappa value (0.636) suggests a good consistency between mNGS and PCR. The specificity of mNGS and PCR was 100%, which was higher than serum BDG. In conclusion, both of mNGS and PCR showed good sensitivity and specificity in accurate diagnosis of PJP in patients infected HIV. However, PCR delivers little value to identify multi-infection, particularly for those uncommon or newly identified pathogens.

In individuals with advanced HIV/AIDS and CD4^+^ T cell counts below 200 cells/ul, in addition to *P. jirovecii*, other opportunistic infections (such as *Mycobacterium*, *Cryptococcus*, *Aspergillus* and *Penicillium marneffei*) as well as mixed infections were also significantly increased at risk (Jiang et al., 2019; Qin et al., 2020; Mao et al., 2022). The wide range of pathogen identification for mNGS enables it to detect mixed infections in PJP patients infected with HIV. Our result demonstrated that 82.83% of PJP patients have detected mixed infections. Notably, we observed other common opportunistic infection pathogen were detected by mNGS, such as *Mycobacterium*, *Penicillium marneffei*, *Aspergillus* and *Parasite*, which are difficult to culture. In addition, for *Aspergillus* and *Penicillium marniffei*, the sensitivity of BALF mNGS was significantly greater compared to that of GM test, and the positive result from GM test cannot distinguish between *Aspergillus* or *Penicillium marniffi* infection. Therefore, our study implied that mNGS may be an effective method for distinguishing mixed infections in HIV-infected patients, especially for rare, novel, and atypical pathogens.

In addition, the mNGS has the potential to offer guidance for effective antimicrobial treatment. Jiang et al. (2021) reported that initial antimicrobial treatment was altered in 71.7% of non-HIV-infected individuals with PJP following the disclosure of mNGS results. In our observation, 86.87% of the PJP patients also modified their initial antimicrobial therapy. Among these adjustments, 37.37% of PJP patients did not receive TMP/SMZ, 6.6% did not initiate caspofungin until the results of mNGS were reported. Notebly, 22.22% of cases received anti-mycobacterial drugs, and 19.19% of cases initiated anti-*Aspergillus* following the identification of *Aspergillus* through mNGS results. Thus, it demonstrated that mNGS is beneficial for accurately diagnosing and properly treating PJP in HIV-infected patients, especially in patients mixed infections. However, it is noteworthy that mNGS alone cannot distinguish pathogens between colonization and infection, as mNGS lacks widely accepted quantitative cutoffs or threshold values. For respiratory infections, common colonizing bacteria such as Veillonella, Prevotella, Rothia, are not consider when interpretation (Jiang et al., 2023). For conditional pathogens, it is necessary to determine whether they are pathogenic bacteria based on their relative abundance and clinical manifestations. If there is a large number of background or miscellaneous bacteria sequences without dominant microorganisms, contamination should be considered first, followed by opportunistic pathogens. Some studies suggested that conditional pathogenic bacteria and fungi, of which the relative abundance is above 30% of the species level, can be considered as potential pathogens (Jiang et al., 2023; Li et al., 2018). For pathogenic pathogens such as Mycobacterium tuberculosis, if the number of reads ≥ 1, it can be considered as pathogenic pathogens (Miao et al., 2018; Jiang et al., 2023; Zhou et al., 2019). In clinical microbiology, determining the clinical significance of microorganisms that may be colonization or contamination is a classic problem, which usually needs to be explained combined with clinical background. Hence, the final diagnosis and prompt treatment of PJP in HIV-infected patients must be based on a systematic analysis of the panorama, including clinical characteristics, abnormal results from other laboratory and radiological examinations, rather than solely relying on mNGS.

The current study has several limitations. Firstly, the sample size of this single center study is small, so biases are inevitable. Secondly, multicenter prospective studies are also necessary. Thirdly, our study did not investigate the impact of the modified antimicrobial treatment in relation to the outcomes based on mNGS outcomes, but only included the improvement of clinical symptoms as the discharge standard for patients. More emphasis should be placed on clinical follow-up in future practice.

## 5 Conclusion

In conclusion, BALF mNGS and real-time PCR are two powerful techniques with high specificity and sensitivity for accurate diagnosis of PJP. Additionally, the benefit of mNGS is that it may identify other organisms besides PJP and it may benefit proper and prompt treatment.

## Data Availability

The raw mNGS data in the study are deposited in the NCBI repository, accession number PRJNA1154800 with the link http://www.ncbi.nlm.nih.gov/bioproject/1154800.

## Footnotes

1
ftp://ftp.ncbi.nlm.nih.gov/genomes/
